# Antitumor Efficacy of Interleukin 12-Transfected Mesenchymal Stem Cells in B16-F10 Mouse Melanoma Tumor Model

**DOI:** 10.3390/pharmaceutics17030278

**Published:** 2025-02-20

**Authors:** Urška Kamenšek, Tim Božič, Maja Čemažar, Urban Švajger

**Affiliations:** 1 Department of Experimental Oncology, Institute of Oncology Ljubljana, SI-1000 Ljubljana, Slovenia; ukamensek@onko-i.si (U.K.); tbozic@onko-i.si (T.B.); 2 Biotechnical Faculty, University of Ljubljana, SI-1000 Ljubljana, Slovenia; 3 Faculty of Health Sciences, University of Primorska, SI-6310 Izola, Slovenia; 4 Department for Therapeutic Services, Slovenian Institute for Transfusion Medicine, SI-1000 Ljubljana, Slovenia; 5 Faculty of Pharmacy, University of Ljubljana, SI-1000 Ljubljana, Slovenia

**Keywords:** mesenchymal stromal stem cells, gene electrotransfer, cell-mediated gene therapy, IL-12 transfection, non-viral gene therapy, in vivo melanoma mouse model, cancer immunotherapy

## Abstract

**Background/Objectives**: Mesenchymal stromal cells (MSCs) hold the potential for tumor-targeted gene delivery due to their ex vivo manipulability, low immunogenicity, scalability, and inherent tumor-homing properties. Despite the widespread use of viral vectors for MSC genetic modification, safety concerns have prompted interest in non-viral alternatives, such as gene electrotransfer (GET). This study aimed to optimize GET parameters for MSCs transfection, assess MSCs biodistribution after in vivo administration, and evaluate the therapeutic potential of interleukin-12 (IL-12)-modified MSCs in a mouse melanoma model. **Methods**: Human MSCs were isolated from umbilical cords under ethically approved protocols. GET protocols were optimized using a fluorescent reporter gene to evaluate transfection efficiency and cell viability. MSC biodistribution was examined following intravenous and intratumoral injections in murine tumor models using luminescent reporter gene. The therapeutic efficacy of IL-12-modified MSCs was assessed in a syngeneic mouse melanoma model. **Results**: Optimized GET protocols achieved a transfection efficiency of 80% and a cell viability of 90%. Biodistribution studies demonstrated effective tumor retention of MSCs following intratumoral injections, whereas intravenous administration resulted in predominant cell localization in the lungs. IL-12-modified MSCs injected intratumorally significantly inhibited tumor growth, delaying tumor progression by five days compared to controls. **Conclusions**: Optimized GET conditions enabled high-efficiency, high-viability MSCs transfection, facilitating their use as effective vehicles for localized cytokine delivery. While the innate tumor tropism of MSCs was not conclusively demonstrated, the study highlights the potential of GET as a reliable non-viral gene delivery platform and underscores the therapeutic promise of IL-12-modified MSCs in tumor-targeted gene therapy.

## 1. Introduction

Mesenchymal stromal stem cells (MSCs) have emerged as promising candidates for cell-mediated cancer gene therapy due to their unique biological characteristics. MSCs were reported to possess the ability to home to tumor sites, driven by interactions between tumor-secreted factors and chemokine receptors on MSCs [1]. This innate tumor-tropic capacity, combined with their low immunogenicity, makes them promising vehicles for the stealthy delivery of therapeutic agents directly to the tumor microenvironment [2,3,4,5]. Additionally, MSCs have great potential for ex vivo expansion and can be isolated from various tissues, including bone marrow, adipose tissue, umbilical cord, and placenta, providing multiple potential sources for therapeutic use [6].

The use of MSCs for cancer gene therapy involves the genetic modification of these cells to produce and deliver therapeutic proteins. The most common approach for genetic modification has historically involved viral vectors, which offer high transfection efficiency and the ability to stably integrate therapeutic genes into the host genome [5,6]. However, the use of viral vectors in clinical settings raises several safety concerns, including the risk of insertional mutagenesis, which may result in oncogene activation and tumorigenesis [7,8]. Additionally, viral vectors are often immunogenic, potentially triggering immune responses that may reduce their efficacy and increase the risk of adverse reactions [7,9]. Furthermore, the complexity and costs associated with good manufacturing practice-compliant large-scale viral vector production pose significant challenges for clinical translation [10].

Given these limitations, non-viral gene delivery methods, such as gene electrotransfer (GET), have gained attraction as safer and potentially more affordable alternatives. GET uses electric pulses to temporarily permeabilize cell membranes, allowing the intracellular delivery of DNA or other genetic materials [11,12,13,14]. This technique is safe, simple, cost-effective, and scalable, making it particularly attractive for clinical applications. Although GET has demonstrated success in various in vivo gene therapy and vaccination applications [15,16,17,18], its use for ex-vivo genetic modification of MSCs requires optimization to balance transfection efficiency with cell viability. Previous studies have shown that high-intensity electric pulses can cause cell damage, reducing viability and compromising MSC function [19,20,21]. Therefore, fine-tuning the electroporation parameters is essential to achieve effective gene transfer while preserving the cells’ therapeutic properties.

In this study, we focused on optimizing GET for the transfection of MSCs with the aim of transfecting them with the cytokine interleukin-12 (IL-12). This therapeutic gene was selected due to its extensively documented and well-established mechanisms underlying its potent antitumor effects. IL-12 promotes immune responses by stimulating the activation and proliferation of T-cells and natural killer cells, enhancing their ability to attack cancer cells [22,23]. Furthermore, IL-12 has been shown to induce the production of interferon-gamma, which plays a key role in orchestrating antitumor immunity [24]. Despite its promising therapeutic potential, the systemic administration of IL-12 is associated with severe toxicities due to its widespread effects on the immune system [24,25,26]. Thus, targeted delivery of IL-12 to the tumor site using MSCs offers a more controlled and localized therapeutic approach, minimizing systemic exposure and reducing the risk of adverse effects.

Following optimization of GET parameters to improve MSCs transfection efficiency, we assessed the MSCs distribution after in vivo administration and therapeutic efficacy of IL-12-expressing MSCs in a well-established B16F10 melanoma model. This model, characterized as immunologically “cold” but highly responsive to IL-12 therapy 45 [27,28,29], was chosen to evaluate both tumor-targeting capability and therapeutic potential.

Our findings question the innate tumor tropism of MSCs. Nevertheless, we confirmed the potential of GET as a non-viral gene delivery platform for MSC-based cancer therapies and highlighted the promise of IL-12-modified MSCs as an effective vehicle for tumor-targeted gene therapy.

## 2. Materials and Methods

### 2.1. MSC Isolation and Characterization

Human umbilical cord-derived MSCs were prepared from allogeneic umbilical cords as a tissue source, according to standard operation protocols approved by the national ethics committee. Umbilical cords were obtained from full-term delivery by Caesarean sections. The tissue from a single donor was initially seeded during ex vivo culture. After initial 7–10 days, the characteristics spindle-shaped adherent cells could be observed, which then began to proliferate extensively and reached appropriate confluence in approximately another 7–10 days, when they were re-seeded (passage 0) at 2000–6000 cells/cm^2^ for further expansion for approximately 5–7 days (passage 1). The cells were then harvested, characterized, and cryopreserved in pre-determined aliquots. For further experiments, an aliquot of MSCs was thawed and cultured for 3–5 days to obtain sufficient number of cells.

MSCs immunophenotyping was performed using the following monoclonal antibodies: FITC-conjugated anti-CD45, anti-CD73, anti-CD90, and anti-CD105; PE-conjugated anti-HLA-DR (all from Miltenyi Biotec, Bergisch Gladbach, Germany). Cell viability was determined using Annexin V-FITC and 7-aminoactinomycin D. Results were analyzed using MACSQuant 10 flow cytometer and MACSQuantify software version 2.13.3 (Miltenyi Biotec) (Supplementary Figure S1). A morphological assessment was performed using an inverted light microscope (Nikon Exlipse TE300, Tokyo, Japan).

### 2.2. Plasmids

Three plasmids were used in this study: pEGFP-N1 (Clontech, Mountain View, CA, USA) reporter plasmid for expression of the green fluorescent protein (GFP) for in vitro transfection efficiency studies, pGL4.51[luc2 CMV Neo] (Promega, Madison, WI, USA) reporter plasmid for expression of the luciferase (Luc) for in vivo tumor tropism and retention studies, and pORFmIL-12 (p40::p35) (InvivoGen, San Diego, CA, USA) plasmid for the expression of mouse IL-12 for in vivo antitumor efficacy study. All three plasmids were transformed into E. coli strain JM109 (Thermo Fisher Scientific, Waltham, MA, USA), amplified in bacterial culture and isolated using the Endo Free Plasmid Mega Kit (Qiagen, Hilden, Germany) according to the manufacturer’s protocol. The purity and concentration of the plasmids were measured spectrophotometrically (Epoch microplate spectrophotometer, Take3 microvolume plate, BioTek, Winooski, VT, USA) and fluorometrically using the Qubit 4 fluorometer (Thermo Fisher Scientific). The final concentrations were adjusted to 1 mg/mL in endotoxin-free water provided with the kit. In addition, the identity and quality of the plasmid were verified by restriction enzyme analysis.

### 2.3. Gene Electrotransfer

MSCs in the exponential growth phase were harvested by trypsinization, centrifuged, and resuspended in electroporation buffer (125 mM sucrose, 10 mM K_2_HPO_4_, 2.5 mM KH_2_PO_4_, 2 mM MgCl_2_·6H_2_O) at a concentration of 25 × 10^6^ cells/mL. For each electroporation, 1 × 10^6^ MSCs were mixed with 10 µL of plasmid DNA, and the mixture was placed between two parallel stainless-steel electrodes with a 2 mm gap. Electroporation was performed using a Cliniporator™ (IGEA S.p.A., Carpi, Italy) or GT-01 pulse generator (Faculty of Electrical Engineering, University of Ljubljana). Following electroporation, MSCs were transferred to 24-well low-attachment plates (Corning Inc., Corning, NY, USA), and incubated for 5 min before adding 1 mL of warm culture medium.

For transfection efficiency tests (GFP imaging and cell viability), the GFP reporter plasmid pEGFP-N1 was used for the transfection of MSCs. In the first round of experiments, standard electroporation conditions used in our laboratory were tested [30]. The protocol utilized a cold (4 °C) electroporation buffer cold (4 °C), and cells were kept on ice throughout the procedure. Four different pulse protocols were tested: GET 1: 8 square wave pulses, 1300 V/cm (260 V), 100 µs pulse duration, 1 Hz; GET 2: 8 square wave pulses, 1300 V/cm (260 V), 100 µs pulse duration, 5000 Hz; GET 3: 8 square wave pulses, 1300 V/cm (260 V), 100 µs pulse duration, 4 Hz; GET 4: 8 square wave pulses, 600 V/cm (120 V), 5000 µs pulse duration, 1 Hz.

In the next round, room temperature conditions were tested. This protocol utilized a warm EP buffer (37 °C) instead of cold, and the cells were not kept on ice. In addition, 100 µL of warm (37 °C) FBS was added to the electroporated cells immediately after transfer to the 24-well low-attachment plate, followed by the addition of media 5 min later [31]. Two different pulse protocols were tested under these conditions: GET 1 and GET 4.

For the in vivo MSCs tracking, MSCs were transfected with luciferase reporter plasmid pGL4.51[luc2 CMV Neo] and for therapeutic efficiency study with the IL-12 plasmid pORFmIL-12 (p40::p35). GET 1 pulses and room temperature conditions were used for the transfection. After electroporation, the cells were transferred to culture flasks and incubated at 37 °C in a humidified incubator with 5% CO_2_.

### 2.4. Lipofection

In parallel to GET, transfection with Lipofectamine 2000 (1 mg/mL solution, Thermo Fisher Scientific) was performed as a comparative transfection method in the transfection efficiency tests. When preparing the cell suspension for GET, part of the cells was resuspended in the medium in a contraction of 6 × 10^3^ cells/mL, and 100 µL (6000 cells) were plated on a black 96-well flat-bottom culture plate (Greiner Bio-One, Kremsmünster, Austria). Lipofection was performed directly on the plate 4 h after plating, when the cells were already attached. The plasmid–DNA–lipid complexes were prepared in OptiMEM medium (Gibco, Waltham, MA, USA) according to the manufacturer’s instructions. After 20 min of incubation at room temperature, 50 µL of the prepared mixture (containing 0.2 µg pDNA and 0.5 µL Lipofectamine) was added dropwise to each well. The transfected cells were incubated in a humidified incubator at 5% CO_2_ and 37 °C until further processing.

### 2.5. GFP Expression Analysis

After electroporation, the cells were diluted to a concentration of 6 × 10^4^ cells/mL and 100 µL (6000 cells) of this cell suspension was transferred to each well of the black 96-well flat-bottom culture plate (Greiner Bio-One, Kremsmünster, Austria) and incubated at 37 °C in a humidified incubator with 5% CO_2_. Two days after transfection, cells were imaged using the Cytation 1 multimodal reader (BioTek Instruments, Winooski, VT, USA) and analyzed using Gen5 data analysis software version 3.11 (BioTek Instruments). Images were acquired using a 4× objective and a monochromator-based optics. Two brightfield images (one out-of-focus image for reference and one defocused image for cell counting) and one GFP fluorescence image (excitation 469 nm, emission 525 nm) were acquired. The laser autofocus was applied, and the exposure settings of the camera were manually optimized and kept the same for each experiment. Images were analyzed using Gen5 data analysis software by masking the defocused bright field image to determine the number of viable cells in a field of view. For the detection of GFP-positive cells, the fluorescence intensity threshold was determined empirically and was kept the same for all experiments. Cells were classified as GFP-positive if the fluorescence signal was detected above the predefined threshold within the extended counting mask. Transfection efficiency was then calculated by dividing the number of GFP-positive cells by the total number of cells in a given field of view.

### 2.6. Cell Viability Assay

After electroporation, the cells were diluted to a concentration of 6 × 10^4^ cells/mL and 100 µL (6000 cells) of this cell suspension was transferred to each well of the black 96-well flat-bottom culture plate (Greiner Bio-One, Kremsmünster, Austria) and incubated at 37 °C in a humidified incubator with 5% CO_2_. Three days after transfection, 10 mL of Presto Blue^®^ viability reagent (Thermo Fisher Scientific) was added to each well. After one hour of incubation in a humidified incubator at 5% CO_2_ and 37 °C, fluorescence intensity was measured using the Cytation 1 Multimodal Reader (BioTek Instruments). The results of cell viability after GET were normalized to the untreated control group.

### 2.7. Mice

Six- to eight-week-old female C57Bl/6NCrl, BALB/cAnNCrl, and Crl:SKH1-Hr^hr^ mice were purchased from Charles River Laboratories (Sant’Angelo Lodigiano, Italy) and subjected to a quarantine and adaptation period of 1 week. During quarantine and experiments, mice were housed under specific pathogen-free conditions at a temperature of 20–24 °C, a relative humidity of 55 ± 10%, and a 12-h light–dark cycle. Food and water were provided ad libitum. Isoflurane gas anesthesia (2–4%, Chiesi, Parma, Italy) was used during tumor cell and MSC injections. The mice were monitored for weight loss and possible side effects throughout the experiment. A tumor volume of approximately 350 mm^3^ or a loss of 20% of body mass were prespecified humane endpoints for the experiments.

### 2.8. Tumor Models Establishment

Tumors were induced by a subcutaneous injection of 100 µL 0.9% NaCl containing 3 × 10^5^ cells into the shaved flanks of syngeneic mice. B16-F10 tumor cells were injected into C57Bl/6 mice, representing an immunologically cold tumor model. To prepare the induced hot tumor model, two B16-F10 tumors were induced on both flanks in C57Bl/6 mice. In addition, CT26 and MC-38 cells, representing immunologically hot tumor models, were injected in BALB/cAnNCrl and Crl:SKH1-Hr^hr^ mice, respectively. After one week, i.e., three days before injection of MSCs, the right tumor was irradiated. During irradiation, the mice were placed in custom-made lead holders with openings for local exposure of the tumors. Only the right tumor was exposed to a single dose of 15 Gy at a dose rate of 1.92 Gy/min, delivered by a Glumay MP1-CP225 X-ray generator (Gulmay Medical Ltd., Surrey, UK) at 200 kV and 9.2 mA with Cu (0.55 mm) and Al (1.8 mm) filtering.

### 2.9. In Vivo MSC Tracking and Retention

Non-invasive bioluminescence imaging was used to visualize the distribution of luciferase transfected MSCs after intravenous and intratumoral injection. When the tumors reached an average volume of 50 mm^3^ (6–7 mm diameter), the mice were randomly divided into different treatment groups with 3–6 animals per group. Luciferase transfection was performed as described. Two days after transfection, the cells were resuspended in 0.9% NaCl. Part of the transfected cells were plated on a black 96-well plate (Greiner Bio-One) to confirm transfection efficiency and determine cell luminescence using the IVIS Lumina XRMS Series III optical imaging system (Revvity, Waltham, MA, USA) (Supplementary Figure S2). The rest of the cells were resuspended to concentration of 2 × 10^7^ cells/mL for intravenous injection and 0.5 × 10^7^ cells/mL for intratumoral injection. Intravenous injection was performed by a tail vein injection of 100 µL of cell suspension containing 2 × 10^6^ of cells using polyethylene tubing and a 29G insulin syringe. To ensure the successful intravenous injection of MSCs and minimize technical errors, we implemented a two-step procedure. First, we confirmed the injection pathway using physiological saline as described in our previously published protocol [32]. Intratumoral injection was performed by injection of 100 µL of cell suspension containing 0.5 × 10^6^ cells directly into the tumor tissue using 29G insulin syringe. The luminescence signal was recorded 15 min after injection of the MSCs and then every day until the signal disappeared using the IVIS Lumina XRMS Series III optical imaging system. At each time point, the bioluminescence signal was measured 15 min after the intraperitoneal injection of 150 µg/g mouse weight of endotoxin-free D-luciferin potassium salt (GoldBio, St Louis, MO, USA) in Endotoxin-Free Dulbecco’s PBS without Ca^2+^ & Mg^2+^ (Sigma Aldrich, St Louis, MO, USA). After intravenous injection, the mice were imaged from both the dorsal and ventral sides.

### 2.10. In Vivo Therapeutic Efficacy

The therapeutic efficacy of IL-12 transfected MSCs was tested in the B16-F10 tumor model. When the tumors reached an average volume of 50 mm^3^ (6–7 mm diameter), the mice were randomly divided into different treatment groups with 5 animals per group. The MSCs were transfected with the IL-12 plasmid as described (GET 1 pulses under room temperature conditions). Two days after transfection, part of the transfected MSCs was used for determination of IL-12 expression using Quantitative Real-Time Polymerase Chain Reaction (Supplementary Table S1). The rest of the transfected cells were collected and resuspended in 0.9% NaCl at a concentration of 0.5 × 10^7^ cells/mL. Depending on the group, the mice were treated with an intratumoral injection of 100 µL of cell suspension containing 0.5 × 10^6^ of IL-12-transfected MSCs, non-transfected MSCs, or Luc-transfected MSCs.

The antitumor effect was determined by measuring three orthogonal diameters (a, b, c) of the tumors with a caliper every second to third day. The tumor volume was calculated using the formula V = a × b × c × π/6. Tumor volumes for each day were interpolated in Excel, and the arithmetic mean values with the standard error of the mean (SEM) were calculated. Tumor growth curves were drawn with error bars representing the SEM. Tumor doubling time was defined as the time at which the tumor volume had doubled from the first day of the experiment. The tumor growth delay was calculated as the difference between the tumor doubling times of the therapeutic group and the control group. In all groups, tumor growth was followed until the tumors reached 350 mm^3^, which represented a humane endpoint.

### 2.11. Statistical Analysis

All in vitro experiments were performed in triplicate. In vivo experiments were repeated twice with 3–5 mice per group unless otherwise stated. Data were analyzed using GraphPad Prism software (version 10.1.2). One-way ANOVA with post hoc multiple comparison tests (Tukey’s test) was used to compare treatment groups. A *p*-value of <0.05 was considered statistically significant.

## 3. Results

### 3.1. Phenotypical and Morphological Assessment of MSCs

**Figure 1:**
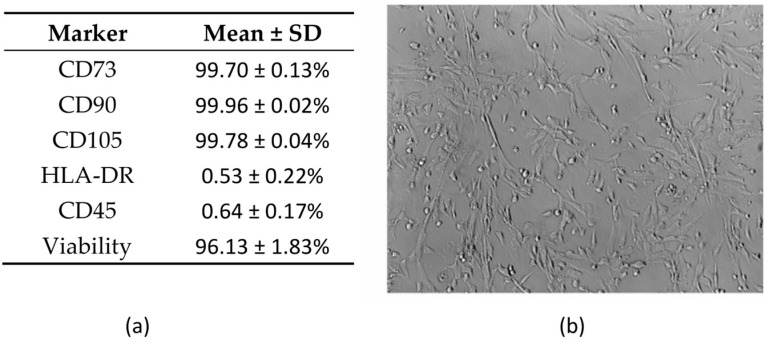
Immunophenotype and morphology of umbilical cord-derived MSCs. (**a**) Flow cytometric analysis data of three independent experiments (three different umbilical cord donors), represented as mean ± SD of percentage of positive cells. (**b**) Morphology of MSCs toward the end of passage 1 of cell culture, displaying typical fibroblast-like morphology. Magnification: ×100.

The great majority of harvested MSCs displayed a strong expression (>99%) of key MSC markers CD73, CD90, and CD105, as well as low or absent expression of CD45 and HLA-DR. Viability was greater than 95% in all cases (Figure 1a, Supplementary Figure S1). The MSCs displayed a predominantly fibroblast-like morphology (Figure 1b).

### 3.2. In Vitro Transfection Efficiency of MSCs Using GET

**Scheme 1:**
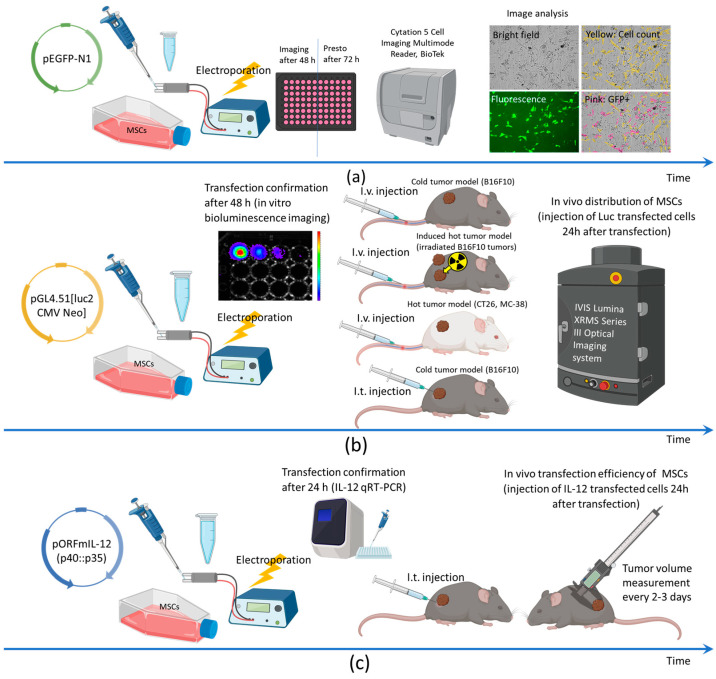
Study design for determination of (**a**) in vitro transfection efficiency (scale bar: 100 µm), (**b**) in vivo distribution, and (**c**) in vivo therapeutic efficiency.

**Figure 2:**
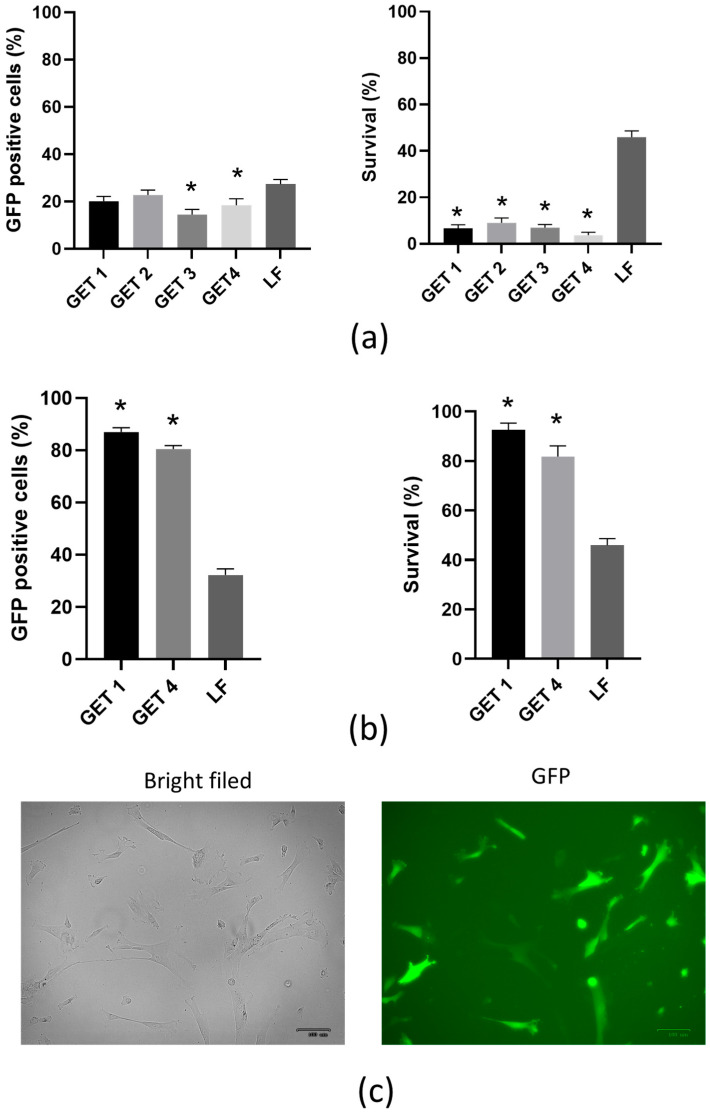
Validation of MSC electroporation protocol. (**a**) Initial testing using four different GET pulses. (**b**) Modified protocol under room temperature conditions using selected GET pulses. (**c**) Fluorescence microscopy image of transfected cells. GET 1 = 8 × 1300 V/cm (260 V/2 mm), 100 µs, 1 Hz; GET 2 = 8 × 1300 V/cm (260 V/2 mm), 100 µs, 4 Hz; GET 3 = 8 × 1300 V/cm (260 V/2 mm), 100 µs, 5000 Hz; GET 4 = 8 × 600 V/cm (120 V/2 mm), 5000 µs, 1 Hz; LF = Lipofection (Lipofectamine 2000); GET, gene electrotransfer; GFP, green fluorescent protein. Results are presented as means of three independent experiments with three replicates ± SEM. Statistical significance: * *p* ≤ 0.05 vs. lipofection group. Scale bar: 100 µm.

The optimization of GET parameters was performed using a GFP reporter gene to determine the transfection efficiency (fluorescence) and cytotoxicity after GET (Scheme 1a). Initial experiments using four different GET pulse protocols demonstrated low levels of GFP reporter gene expression (around 20%) with a significantly lower cell viability (around 10%) compared to the lipofection method (Figure 2a). By eliminating the use of ice baths, a significant improvement in both transfection efficiency and cell viability was observed. Under the room temperature conditions, transfection efficiency increased to 80%, and cell viability was improved to approximately 90% using the GET 1 pulses (Figure 2b). Transfection efficiency and cell viability after both tested GET pulses were significantly higher than after lipofection. The successful transfection of MSCs was also confirmed by fluorescence microscopy, with GFP expression clearly visible in the majority of cells (Figure 2c). Room temperature conditions and GET 1 pulses were therefore selected for the transfection of MSCs in succeeding in vivo tests for transfection of the reporter luciferase and the therapeutic IL-12 gene.

### 3.3. In Vivo Distribution and Tumor Retention of MSCs

To assess the distribution and retention of MSCs in vivo, MSCs were transfected with the luciferase plasmid using the selected GET protocol (room temperature conditions, GET 1 pulses) and 24 h later injected into tumor-bearing mice either intravenously or intratumorally (Scheme 1b). Before the injection, the cells’ luminescence (transfection efficiency) was confirmed using bioluminescence imaging (Supplementary Figure S2).

**Figure 3:**
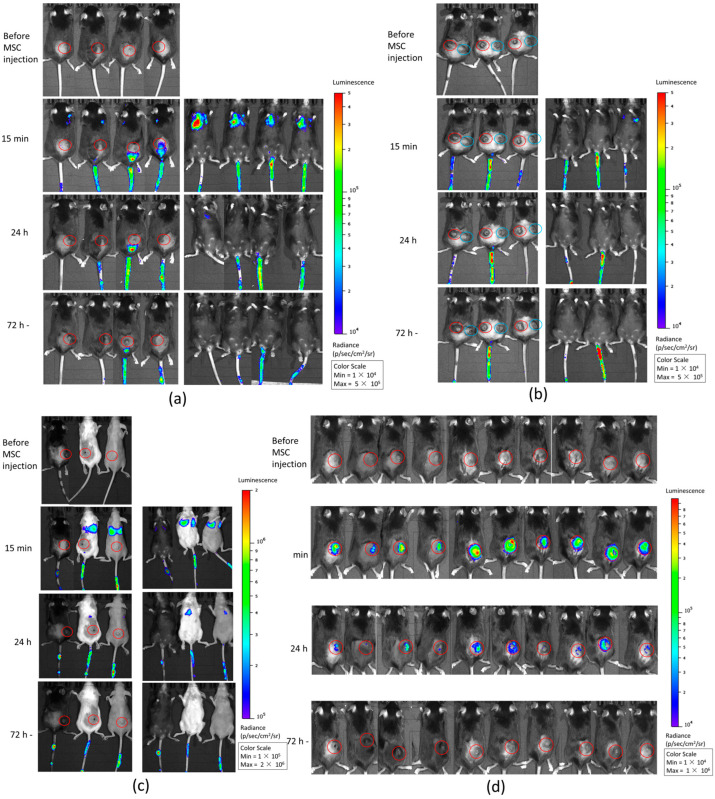
In vivo tracking of MSCs. Distribution of Luc-transfected MSCs after intravenous injection in (**a**) immunologically cold tumor model B16F10, (**b**) induced hot tumor model, i.e., irradiated B16-F10 tumors) and (**c**) immunologically hot tumor models CT26 and MC-38. (**d**) Retention of Luc-transfected MSCs after intratumoral injection in B16F10 tumor model. Location of tumors is marked with circles. In the dual tumor model (**b**), irradiated tumors are marked with red circles and unirradiated tumors with blue. Images of all included mice are presented; 3–4 mice were included for the intravenous injections, and 10 mice in two independent experiments for intratumoral injections. Luminescent signal is presented as radiance (photons/s/cm^2^/sr) in a logarithmic rainbow scale. To facilitate better comparison, the same radiance range was applied to all images.

Following intravenous injection, whole body in vivo bioluminescence imaging revealed that MSCs predominantly localized in the lungs and at the injection site (Figure 3a–c). This pattern was consistent across all tumor models: the immunologically cold B16-F10 (Figure 3a), the induced hot tumor model, i.e., irradiated B16-F10 tumors (Figure 3b), and also intrinsically hot tumor models CT26 and MC-38 (Figure 3c).

In contrast, MSCs injected intratumorally were retained at the tumor site for up to 2 days post injection (Figure 3d). The luminescent signal remained localized within the tumor mass, with no detectable spread to surrounding tissues.

### 3.4. In Vivo Therapeutic Efficacy of IL-12-Transfected MSCs

**Figure 4:**
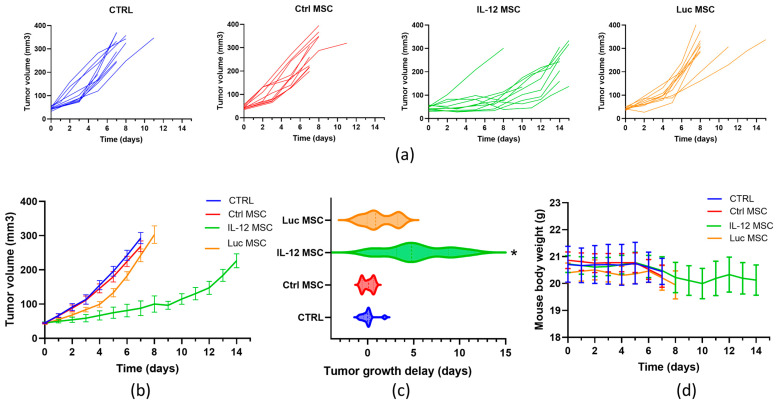
Therapeutic efficacy of IL-12-transfected MSCs: (**a**) Tumor growth curves for individual mice. (**b**) Average tumor growth curves drawn as mean tumor volume with standard error of mean (**c**) Tumor growth delay (difference between tumor doubling times in control and therapeutic groups) drawn as violin plot with mean. (**d**) Average mice body weight drawn as mean body weight with standard error of the mean. CTRL = untreated tumor-bearing mice, Ctrl MSC = mice treated with intratumoral injection of non-modified MSCs; IL-12 MSC = mice treated with intratumoral injection of IL-12-transfected MSCs: Luc MSC = mice treated with intratumoral injection of Luc-transfected MSCs. Two independent experiments with 5 animals per group. Statistical significance: * *p* ≤ 0.05 vs. all groups.

The therapeutic efficacy of MSCs transfected with IL-12 was evaluated in mouse melanoma model B16-F10 (Scheme 1c). The expression of IL-12 from transfected MSCs was first confirmed by qRT-PCR (Supplementary Table S2). Mice were treated with intratumoral injection of either IL-12-transfected MSCs, non-modified MSCs, or Luc-transfected MSCs. The group treated with IL-12-MSCs showed significant tumor growth inhibition compared to the nontreated control, the group treated with non-modified MSCs and also the group treated with Luc-transfected MSCs (Figure 4a,b). The treatment was well tolerated, as evident from mice body weight showing no significant weight loss (Figure 4c). The IL-12-MSC group exhibited a tumor growth delay of approximately 5 days (Figure 3d). The non-modified MSC group did not show a significant tumor growth delay compared to the control group (Figure 4b).

## 4. Discussion

In this study, we successfully optimized GET for the non-viral genetic modification of MSCs, establishing an effective approach to use MSCs as carriers for cancer gene therapy. Several viral as well as non-viral methods for MSC genetic modification have already been evaluated for various cancer and non-cancer-related applications [33,34,35,36,37]. For non-immortalized MSCs to serve as effective gene therapy vectors, certain key attributes are essential. While stable integration of the transgene, achievable with viral vectors, is not required and is even unwanted when working with non-immortalized cells, high transgene transfer efficiency and preservation of MSCs’ robust secretory capacity are crucial, particularly for delivering anticancer cytokines to the tumor microenvironment [10]. Additionally, maintaining MSC viability and low immunogenicity is vital for the stealthy delivery of modified MSCs. Practicality and cost-effectiveness are also important considerations, especially for scaling up clinical production levels. Electroporation, a physical approach for introducing non-viral vectors, meets all these requirements. Previous studies indicated that MSCs require finely tuned electroporation parameters due to their sensitivity to cellular damage [21,38,39,40]. However, with optimized electroporation protocols, high transfection efficiency could be achieved, even when working with large quantities of MSCs [40].

Optimizing the GET protocol was, therefore, the first step in our experimental design. Initial experiments under our standard GET conditions normally used for the transfection of tumor cells [30] resulted in low transfection efficiency and very poor cell viability of MSCs, regardless of the electroporation pulses used. This indicated that these conditions were too harsh for MSCs. One factor that could enhance the viability was the temperature. We figured that electroporating MSCs at room temperature, as opposed to ice-cold conditions, could reduce stress and also enhance membrane fluidity, therefore improving both cell viability as well as transfection efficiency. Studies have indicated that conducting electroporation at moderate temperatures supports better membrane resealing, allowing for efficient plasmid uptake while preserving cellular integrity [41,42]. This aligns with our own findings, where shifting to warm electroporation buffer and room temperature conditions resulted in significantly improved transfection outcomes and overall cell survival with both tested electroporation pulses. These adjustments highlight that electroporation protocols tailored to temperature can be especially beneficial for sensitive cells like MSCs, where maintaining functionality is paramount. The optimized protocol, incorporating these temperature adjustments, achieved a transfection efficiency of 80% and cell viability of approximately 90% and was therefore selected for the succeeding in vivo distribution and therapeutic efficacy tests.

Compared with lipofection, a common chemical-based transfection method, the optimized GET method achieved superior transfection efficiency and viability. Lipofection, though effective in some cell types, is generally associated with lower efficiency in primary cells [35,39]. By contrast, GET avoids the use of chemical agents and offers a less cytotoxic approach for the transfection of MSCs. These results support growing evidence that, with optimization, physical transfection methods can achieve both high transfection rates and maintain cell integrity [19,20,21,40], positioning GET as a safer, cost-effective option for clinical application.

One of the most compelling attributes of MSCs for targeted gene therapy is their reported tumor-homing capability [1,43,44]. Direct evidence of MSC tropism toward tumor and wound microenvironments has been demonstrated through various detection techniques, including in vivo bioluminescent imaging [1,43]. MSCs are naturally attracted to sites of inflammation, injury, and necrosis, which share similarities with the tumor microenvironment. However, in our experiments, MSCs administered systemically tended to localize predominantly in the lungs rather than at the tumor site in the B16F10 melanoma model. This tendency could be partially explained by the “cold” nature of the B16F10 melanoma, which is defined by low immune cell infiltration, an immunosuppressive microenvironment, and reduced MHC class I expression [27,28,29]. These features render the tumor less detectable to the immune system and may make it less attractive to MSCs.

To examine whether a more immune-active tumor might enhance MSC homing, we additionally tested the homing in two immunologically “hot” tumor models, CT26 and MC-38 [28,29,45,46,47]. Additionally, we explored the potential of enhancing MSC recruitment by irradiating B16F10 tumors to induce inflammation and necrosis [44,48,49,50]. Nonetheless, even with these modifications, MSCs administered systemically continued to accumulate mainly in the lungs rather than at the tumor sites in both hot and irradiated models.

This pulmonary entrapment of MSCs following systemic administration is consistent with previous studies showing that MSCs often remain in the pulmonary circulation soon after injection, limiting their effectiveness in targeting tumors located outside the lungs [24,51,52,53]. Although previous studies have reported MSC tumor homing in specific conditions or models [1,43,54], our findings support the notion that systemic MSC delivery generally lacks sufficient targeting efficacy, purely by merit of their innate tropism efficacy. For a definitive assessment of MSC tropism, larger animal models and non-xenogeneic MSCs could provide more conclusive results. To overcome the limitations of systemic delivery, we explored intratumoral injection, which retained MSCs at the tumor site for several days. This supports intratumoral injection as a preferred method for therapies that require sustained expression within the tumor microenvironment, such as IL-12 used in our study.

Our study adds to the existing research supporting IL-12-modified MSCs for cancer therapy [25,50,51,54,55,56,57,58,59,60,61,62,63,64]. IL-12 is a potent immunostimulatory cytokine with well-documented antitumor effects, though its clinical application has been limited by toxicity associated with high systemic doses and by its short half-life. Localized IL-12 therapy offers a promising approach, aiming to leverage its potent immunostimulatory properties while avoiding systemic toxicity. This strategy aligns with findings from other IL-12 studies, suggesting that local delivery methods, such as in situ gene therapy by GET, can significantly reduce systemic toxicities while maximizing therapeutic efficacy at the tumor site [24,25,65,66]. In our study, IL-12-transfected MSCs delayed melanoma progression by five days compared to controls, demonstrating IL-12’s therapeutic potential when delivered locally. Similar positive therapeutic outcomes have been previously reported using virally IL-12-transduced MSCs in various local and metastatic tumor models, including melanoma, glioblastoma, and mammary, hepatocellular, lung, colon, and renal cell carcinoma [10,25,50,51,53,54,55,56,57,58,59,60,61,62,63,64]. Together, these studies showed that IL-12-modified MSCs prolonged survival and induced immune cell recruitment and an overall favorable immune environment within the tumor.

In the most recent study with the B16F10 mouse melanoma model, Kulach et al. used mouse MSCs isolated from the bone marrow and genetically modified them to express IL-12 through viral transduction [55]. The authors investigated the tumor tropism of these MSCs in vitro and evaluated their therapeutic efficacy in vivo in both subcutaneous tumors and lung metastases. Interestingly, although their in vitro experiments confirmed the tumor tropism of MSCs, their in vivo approach employed intratumoral injections in the subcutaneous tumor model, likely to ensure MSCs delivery to the tumor site, while intravenous injections were reserved for the lung metastasis model. The study demonstrated that this strategy effectively reduced tumor burden in both primary and metastatic melanoma settings.

The importance of MSCs presence within the tumor was first documented by Seo et al., who demonstrated that intratumoral injection of IL-12-MSCs produced greater antitumor effects than systemic injection in B16F10 melanoma model [51]. Numerous studies have since favored localized over systemic administration of IL-12-MSCs to treat various tumors [50,52,55,57,60,64,67]. For instance, Hu et al. reported that MSCs transfected with IL-12 using nanoparticles effectively inhibited B16F10 lung metastases but not subcutaneous B16F10 tumors following intravenous administration; however, IL-12-MSCs were effective against subcutaneous tumors when administered intratumorally [52]. The best results across studies emerged from repeated treatments or combination therapies, such as combining IL-12-MSCs with PD-1 blockade [37,55,67]. These findings support the potential of IL-12-modified MSCs for broad cancer therapy applications when administered locally.

The limited tumor-targeting ability of MSCs demonstrated in our and other studies remains a challenge. While intratumoral injection provides an effective delivery method for accessible tumors, systemically administered MSCs require improved targeting to reach distant or metastatic sites. Engineering MSC surface receptors to enhance tumor homing or using smaller carriers like exosomes could offer solutions [68,69,70]. Additionally, combination therapies could improve the efficacy of MSC-based IL-12 delivery. For example, using local ablative therapies like radiotherapy or immune checkpoint inhibitors in combination with IL-12-MSCs could enhance immune responses within the tumor [37,50,55,67]. Combining IL-12 delivery with checkpoint blockade has already shown promising results in preclinical studies, enhancing antitumor immunity and providing durable responses in glioblastoma models [55,67]. Furthermore, as GET can deliver larger genetic payloads, simultaneous transfer of multiple therapeutic genes is possible [8,71,72]. This capability could allow for complex modifications of MSCs, transforming them into stealthy effector cells that can identify and target cancer cells throughout the body.

One of the significant attributes of MSCs is their low inherent immunogenicity, allowing for allogeneic and even xenogeneic applications [73,74]. In our study, human MSCs introduced into murine models did not induce adverse reactions, supporting their low immunogenic profile. Even in mice where MSCs accumulated in the lungs, no signs of discomfort were observed, and animals were ultimately sacrificed due to tumor size rather than any MSC-related effects. It is worth mentioning that we used umbilical cord-derived MSCs, which are recognized for their exceptionally low immunogenicity [73]. Their neonatal origin also makes them less prone to pro-tumorigenic interactions, which could otherwise support tumor progression or therapy resistance [75,76,77]. These findings emphasize the dual role of MSCs in the tumor microenvironment, where their therapeutic potential must be carefully balanced against their capacity to contribute to tumor progression and resistance mechanisms.

Given the inherently low immunogenicity of umbilical cord-derived MSCs, it is particularly important to minimize any increase in their immunogenic profile during genetic modification [10]. Using plasmid vectors is advantageous in this respect, as they generally induce lower immunogenicity than viral vectors [7,8,35]. However, immunogenicity can also depend on the transgene itself. For example, luciferase, used in this study and in many others to track MSCs distribution [1,43], is inherently immunogenic [78] and likely contributed to the premature clearance of Luc-transfected MSCs from tumors observed in our study Future studies could consider alternative markers for MSC tracking without enhancing immunogenicity, independently of the transgene used.

An advantage of GET-based genetic modification of MSCs used in our study is its inherent safety compared to viral methods. Although viral vectors are highly efficient, they carry risks such as insertional mutagenesis, which may lead to long-term pro-tumor effects, high immunogenicity complicating repeated dosing, and manufacturing, as well as regulatory complexities that limit clinical utility [3,5,6,10,79]. Non-viral methods like GET address these issues, offering a safer, potentially more cost-effective alternative. The transient nature of non-immortalized MSCs also introduces a built-in safety measure, as these cells naturally clear over time, particularly in an allogeneic setting [10]. The low immunogenicity associated with GET allows for repeated treatments, providing better control over transgene expression duration and reducing the risk of long-term side effects, aligning with safety guidelines for gene therapy applications.

Advancements in GET technology further support its clinical potential for MSC-based therapies. Improvements in vector design are progressing from conventional plasmids to smaller, synthetic DNA or RNA constructs [80,81,82,83,84]. Meanwhile, electroporation devices now offer better pulse parameter control, reducing cytotoxicity and enhancing reproducibility across cell types. These improvements support adherence to good manufacturing practice standards, which are essential for clinical translation [85]. Devices such as CliniMACS Prodigy^®^ (Miltenyi Biotec), MaxCyte^®^ electroporation systems (MaxCyte, Rockville, MD, USA), and Flowfect^®^ (Kytopen, Cambridge, MA, USA), primarily developed for CAR-T cell production, enable scalable, reproducible MSC transfection, bridging the transition from research to clinical application for MSC-based gene therapies.

Despite the promising outcomes of this study, several limitations should be acknowledged. Firstly, since IL-12 was used as a model therapeutic gene with well-established antitumor mechanisms, we chose not to reinvestigate these aspects in our study, also adhering to the 3R principles of animal experimentation. However, we recognize that MSC-mediated delivery of IL-12 may introduce additional immunological effects that warrant further investigation in future studies. Secondly, the systemic delivery of MSCs demonstrated limited tumor-homing efficiency, with most cells being trapped in the pulmonary circulation rather than localizing to the tumor site. This restricts their therapeutic potential for targeting distal or metastatic tumors. Additionally, while our optimized GET protocol achieved high transfection efficiency and cell viability, the transient nature of non-integrating plasmids may require repeated treatments to maintain therapeutic efficacy, potentially increasing the complexity and cost of clinical translation. Furthermore, the use of human MSCs in murine models, although informative, may not fully recapitulate the biological interactions in a homologous system, necessitating validation in larger animal models or human clinical trials. Lastly, the immunogenicity of the luciferase reporter gene used for cell tracking may have contributed to premature MSC clearance, highlighting the need for alternative, less immunogenic tracking methods. Addressing these limitations in future studies will be critical for advancing MSC-based gene therapies toward clinical application.

## 5. Conclusions

This study demonstrates the effective use of GET for the non-viral genetic modification of MSCs to create a potential therapeutic tool for cancer treatment. By optimizing GET, we achieved high transfection efficiency and cell viability, allowing MSCs to serve as carriers for localized IL-12 therapy that effectively delays tumor growth in a melanoma model. While our results call into question the innate tumor tropism of MSCs, they underscore the promise of GET as a robust, non-viral gene delivery platform and highlight the therapeutic potential of IL-12-modified MSCs for tumor-targeted gene therapy.

## Data Availability

The data presented in this study are available on request from the corresponding author.

## Supplementary Materials

The following supporting information can be downloaded at: https://www.mdpi.com/article/10.3390/pharmaceutics17030278/s1, Table S1: List of primers used for qRT-PCR; Table S2: Determination of mIL-12α expression in transfected MSCs by qRT-PCR; Figure S1; Representative flow cytometry analysis for immunophenotyping of MSCs; Figure S2: Transfection confirmation by in vitro bioluminescence imaging.
