# Supplementary material for: Antitumor Efficacy of Interleukin 12-Transfected Mesenchymal Stem Cells in B16-F10 Mouse Melanoma Tumor Model
Source: Pharmaceutics. 2025 Feb 20;17(3):278. doi: 10.3390/pharmaceutics17030278 (PMC11944637; doi:10.3390/pharmaceutics17030278)
Supplement: Supplementary file 1 [file pharmaceutics-17-00278-s001.zip › pharmaceutics-3417134-supplementary.pdf]

pharmaceutics

Supplementary materials

Article

Antitumor Efficacy of Interleukin 12-Transfected Mesenchymal Stem Cells in B16-F10 Mouse Melanoma Tumor Model

Urška Kamenšek [1,2], Tim Božič [1,2], Maja Čemažar [1,2,3,*] and Urban Švajger [2,4,*]

Supplementary Table S1: List of primers used for qRT-PCR. 24 hours after the transfection cells were lysed and total RNA was isolated using Total RNA Kit, peqGOLD (VWR) according to the manufacturer's instructions. Then, first strand cDNA was generated from 2 μg of template RNA using a Thermocycler Primus 25 (Peqlab, UK) and SuperScript VILO cDNA Synthesis Kit (Thermo Fisher Scientific) in a 20 μL reaction mix, according to the manufacturer's instructions. qRT-PCR was performed with QuantStudio 3 Real-Time PCR System (Thermo Fisher Scientific). The samples were prepared using SYBR PowerUp Master Mix (Thermo Fisher Scientific) and predesigned specific primers (IDT, IA, US). The standard thermocycling program consisted of a 95°C denaturation for 2 min, followed by 40 cycles of 95°C for 15 s and 60°C for 60 s. All samples were run in triplicates and the products were analyzed on QuantStudioTM 3 Real-Time PCR System (Thermo Fisher Scientific). Reactions with the template free control were included for each set of primers.

Target	Species	Sequence (5'-3')
*E2F1*_F	Human	CCAGGAAAAGGTGTGAAATC
*E2F1*_R	Human	AAGCGCTTGGTGGTCAGATT
*GUSB*_F	Human	AGGTGATGGAAGAAGTGGTG
*GUSB*_R	Human	AGGATTTGGTGTGAGCGATC
*Il-12α*_F	Mouse	CGGCAGCAGAATAAATATGAG
*Il-12α*_R	Mouse	GAGTTCTTCAAAGGCTTCATC

Supplementary Table S2: Determination of mIL-12α expression in transfected MSCs by qRT-PCR. The table presents the average Ct values for mouse IL-12 (mIL-12) and human housekeeping genes E2F transcription factor 1 (*E2F1)* and glucuronidase beta (*GUSB)* in the control and GET-transfected MCSs.

Average Ct Values	hE2F1	hGUSB	mIL-12α
Ctrl	26.05	25.10	37.23
GET	26.71	24.93	20.06

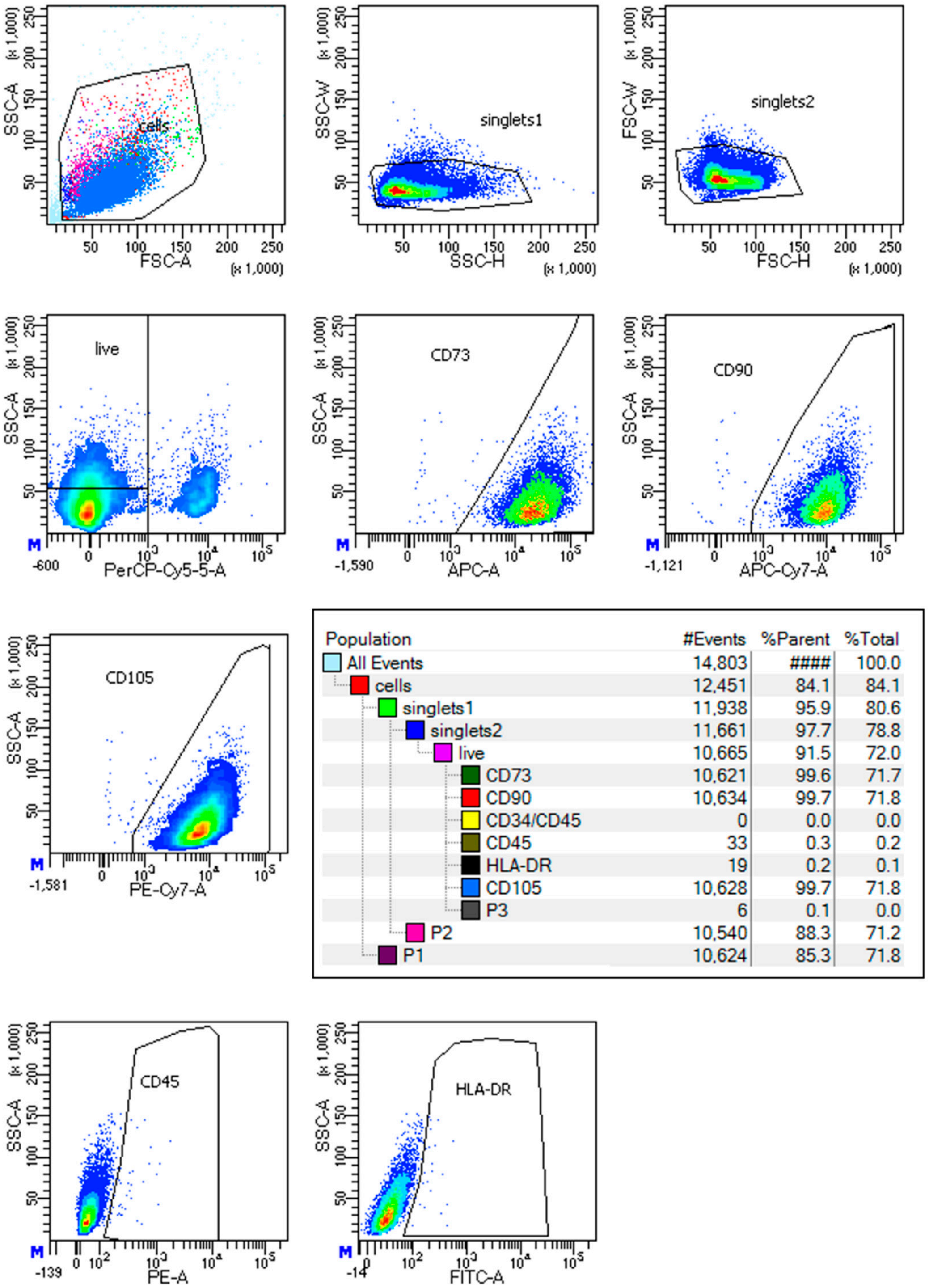

Population	#Events	%Parent	%Total
All Events	14,803	####	100.0
cells	12,451	84.1	84.1
singlets1	11,938	95.9	80.6
singlets2	11,661	97.7	78.8
live	10,665	91.5	72.0
CD73	10,621	99.6	71.7
CD90	10,634	99.7	71.8
CD34/CD45	0	0.0	0.0
CD45	33	0.3	0.2
HLA-DR	19	0.2	0.1
CD105	10,628	99.7	71.8
P3	6	0.1	0.0
P2	10,540	88.3	71.2
P1	10,624	85.3	71.8

Supplementary Figure S1: Representative flow cytometry analysis for immunophenotyping of MSCs. Following monoclonal antibodies were used: FITC-conjugated anti-CD45, anti-CD73, anti-CD90 and anti-CD105; PE-conjugated anti-HLA-DR (all from Miltenyi Biotec, Bergisch Gladbach, Germany). Cell viability was determined using Annexin V-FITC and 7-aminoactinomycin D. Results were analyzed using MACSQuant 10 flow cytometer and MACSQuantify software (Miltenyi Biotec).

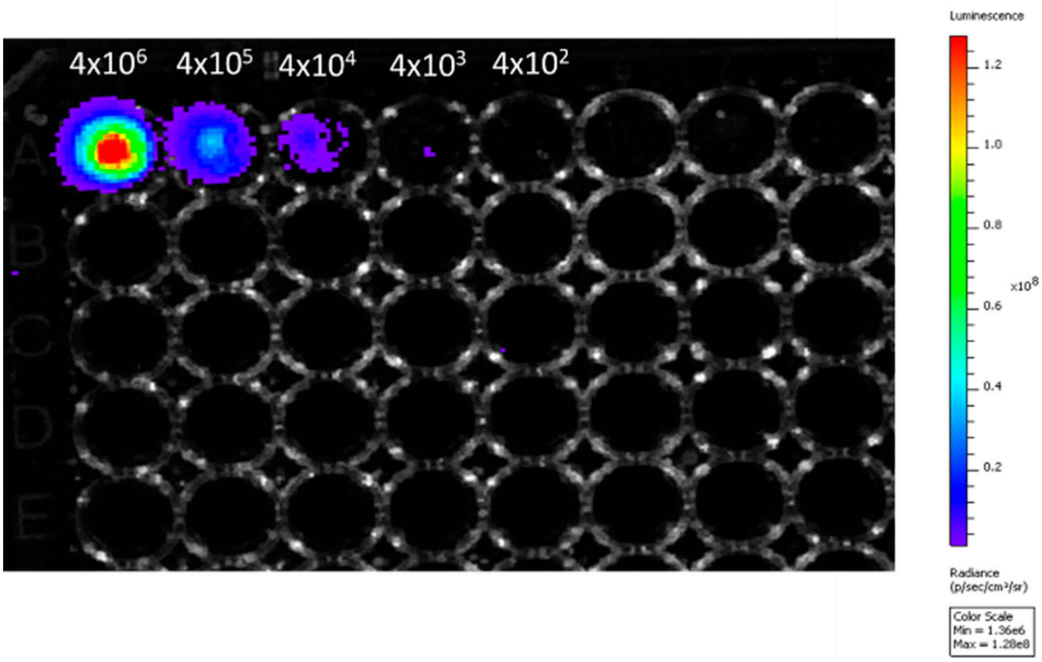

Supplementary Figure S2: Transfection confirmation by in vitro bioluminescence imaging. 24 hours after the transfection, serial dilutions of cells ranging from 4×10^6, 4×10^5, 4×10^4, 4×10^3 and 4×10^2 cells were plated on a black 96-well plate (Greiner Bio-One). Luciferin (D-Luciferin, Potassium Salt, Invitrogen) was added to the cells at a concentration of 150 µg/ml and luminescence was measured using the IVIS Lumina XRMS Series III optical imaging system. Luminescent signal is presented as radiance (photons/s/cm^2/sr) in a rainbow scale.
